# Supplementary figures and images for: Effects of thermal stress on amount, composition, and antibacterial properties of coral mucus
Source: PeerJ. 2019 Apr 29;7:e6849. doi: 10.7717/peerj.6849 (PMC6497039; doi:10.7717/peerj.6849)

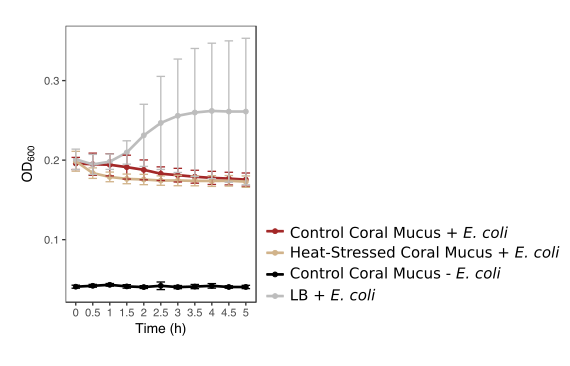

Supplement: Figure S1 — Optical density (OD) at 600 nm indicates the density of bacteria in coral mucus or technical control samples. Red lines represent mucus from corals under the control condition with added E. coli. Beige lines represent mucus from heat-stressed fragments with added E. coli. The black line represents a negative control of coral mucus from healthy coral fragments without added E. coli. The grey line represents a positive control of bacterial growth media (LB) with added E. coli. Error bars represent standard error. [file peerj-07-6849-s001.png]
